# Cost consequences of task-shifting intravitreal injections from physicians to nurses in a tertiary hospital in Norway

**DOI:** 10.1186/s12913-023-09186-0

**Published:** 2023-03-08

**Authors:** Stine Bolme, Dordi Austeng, Tora Sund Morken, Turid Follestad, Vidar Halsteinli

**Affiliations:** 1 Department of Ophthalmology, St. Olavs Hospital, Trondheim University Hospital, Trondheim, Norway; 2 Department of Neuromedicine and Movement Science, Norwegian University of Science and Technology, NTNU, Trondheim, Norway; 3 Department of Clinical and Molecular Medicine, Norwegian University of Science and Technology, 7491 Trondheim, Norway; 4 Regional Centre for Health Care Improvement, St. Olavs Hospital, Trondheim University Hospital, Trondheim, Norway

**Keywords:** Task-shifting, Physicians versus nurses, Anti-VEGF injections, Hospital costs, Societal costs, Cost projections

## Abstract

**Background:**

Anti-vascular endothelial growth factor is a medicine administered intravitreally by an injection to maintain visual acuity in patients with a variety of retinal diseases. The demand for this treatment has grown considerably in the westernized world the last two decades and will continue to increase due to an aging population. Because of the high volume, injections seize enormous resources and represent high costs for both hospitals and society. Task-shifting of injections from physicians to nurses may be a means to reduce such costs, however the magnitude of possible savings has been poorly investigated. To this end we investigated changes in the hospital costs per injection, six-year cost projections of physician- versus nurse-administered injections for a Norwegian tertiary hospital and we compared the societal costs per patient per year.

**Methods:**

Patients (*n* = 318) were randomized to either physician- or nurse administered injections, and data were prospectively collected. Hospital costs per injection were calculated as the sum of training costs, personnel time and running expenses. The number of injections for the years 2014 – 21 from a Norwegian tertiary hospital was combined with age group specific injection prevalence and population projections to calculate cost projections for 2022 – 27. Societal costs per patient were calculated as the sum of hospital costs, transport costs for patients, caregivers’ use of time, costs of ophthalmology consultations and community-based homecare.

**Results:**

The hospital costs per injection were 5.5 € higher for physicians compared to nurses (281.6 € versus 276.1 €). Cost projections estimated an annual hospital saving of task-shifting of 48 921 € for 2022 – 27. Societal costs per patient did not differ significantly between the two groups (mean 4988 € vs 5418 €, *p* = 0.398).

**Conclusion:**

Task-shifting of injections from physicians to nurses can reduce hospital costs and increase the flexibility of physician resources. The annual savings are modest, but increased demand for injections might increase future cost savings. To achieve future savings for society, organizing ophthalmology consultations and injections on the same day to reduce the number of visits might be a solution.

**Trial registration:**

ClinicalTrials.gov NCT02359149 (09/02/2015).

## Introduction

Injections with anti-vascular endothelial growth factor (anti-VEGF) have become the most common intraocular procedure worldwide due to its considerable effect on several retinal diseases such as age-related macular degeneration (AMD), retinal vein occlusion (RVO) and diabetic macular oedema (DMO) [1]. The chronic nature of these diseases results in repetitive, often life-long treatment. With an ageing population, managing this fast-growing patient pool will occupy an increased amount of physician resources and cause increased healthcare costs [2, 3]. A randomized controlled trial from Norway has reported that task-shifting injections from physicians to nurses resulted in similar visual function and safety profile [4]. However, the economic consequences of task-shifting were not investigated. This paper aimed to assess economic consequences from a Norwegian tertiary hospitals’ perspective with respect to hospital costs per injection and potential future cost savings, and in addition societal costs per patient. The hypothesis was that task-shifting would lower costs.

## Methods

318 patients were recruited as part of a randomized controlled trial examining safety of task-shifting injections from physicians to nurses. Further description of the recruitment and randomization of patients has earlier been published [4]. The study was conducted at St. Olavs Hospital (StO-study), Trondheim University Hospital, serving 300.000 inhabitants in Central Norway, and data were prospectively collected from March 2015 to May 2017. Inclusion criteria were having either AMD, RVO or DMO eligible for anti-VEGF treatment. In both groups, the mean inclusion time in the StO-study was 50 weeks (SD_physician_ = 9.1, SD_nurse_ = 8.1). The patient baseline characteristics are shown in Table 1, in addition to service utilization in terms of number of eyecare consultations and injections. Patients received between one and 12 injections, according to their treating physician's discretion.

**Table 1:** Baseline and demographic characteristics of randomized patients

	Physician	Nurse
	*n* = 155	*n* = 163
Mean age, years (SD)	75 (11)	75 (10)
Female, no. (%)	75 (48)	88 (54)
Eyecare (no. of consultations per patient) (SD)	5.2 (2.2)	(5.3) (2.6)
No. of injections (SD)	6.9 (2.8)	6.9 (2.6)
Median living distance from hospital, km (25–75 pct)	33 (7—93)	38 (9—90)
**Degree of independence:**		
Homecare, mean minutes per week (SD)	19 (58)	20 (85)
Depending on help in daily living^a^, no. (%)	36 (23)	38 (23)
Patients traveling with caregiver, no. (%)	44 (28)	57 (35)
**Work situation:**		
Retired, no. (%)	131 (85)	135 (83)
Paid work, no. (%)	16 (10)	18 (11)

^a^Lives with relatives, lives in a nursing home, lives in municipal care home

Detailed cost calculations were performed, which included the application of a range of unit costs (Table 2). The basic principle was first to identify the type and volume of cost elements and services and next multiply by the relevant unit cost. Of specific importance was the wage difference between physicians and nurses. Each personnel group’s mean annual gross income, including social expenses in 2017, was divided by the number of hours working that year (1748 h for physicians and 1725 h for nurses) to find the wage cost per hour. A detailed description of the costs is given below. Costs were measured in euros (€). Norwegian kroner (NOK) were converted using the rate 1 €^2^◦¹^7^ = 9.3295 NOK^2^◦¹^7^. All costs were adjusted to represent costs in 2017 [5].

**Table 2:** Unit costs. Identification, quantification, and valuation of the unit costs used in the calculations

Cost categories	Unit	Valuation	Unit price
**Hospital costs**^**a**^			
Consultant	Hour	Wage	€ 84
Physician	Hour	Wage	€ 61
Certified injection nurse	Hour	Wage	€ 47
Nurse in training	Hour	Wage	€ 45
Secretary	Hour	Wage	€ 36
Injection medicine	Injection	Real cost	€ 198
Disposable equipment	Injection	Real cost	€ 8
Reusable equipment	Injection	Real cost	€ 11
Cleaning per m^2^	Month	Real cost	€ 6
Renting location per m^2^	Month	Real cost	€ 83
Electricity per m^2^	Month	Real cost	€ 2
**Other healthcare costs**			
Eyecare^**b**^	Visits	Estimated cost	€ 148
Homecare nurse^**c**^	Hour	Charge	€ 90
Homecare other personnel^**c**^	Hour	Charge	€ 87
**Transport costs**^**d**^			
Self-owned car	Km	Real cost	€ 0.2
Taxi	Km	Real cost	€ 1
Public transport (bus)	One-way ticket	Estimated cost	€ 8
Caregivers use of time	Hour	Real cost	€ 23

^**a**^ Source of information: St. Olavs Hospital

^**b**^ Source of information: The tariff booklet for consultation prizes

^**c**^ Source of information: Trondheim municipality

^**d**^ Source of information: Patient travel Norway, Singel Ticket fares (AtB) and Statistics Norway

### Hospital costs per injection

We estimated hospital costs per injection administered by physicians and nurses, respectively. Hospital costs included costs of training, time cost for personnel, clinical support given by a consultant (experienced ophthalmologist), secretary and other support personnel, equipment including medicine, and running expenses of the premises. While the three first cost categories (training, time cost and clinical support) differ between physicians and nurses, the last three were fixed costs and equal for both personnel groups.

#### Training costs

**Fig. 1:**
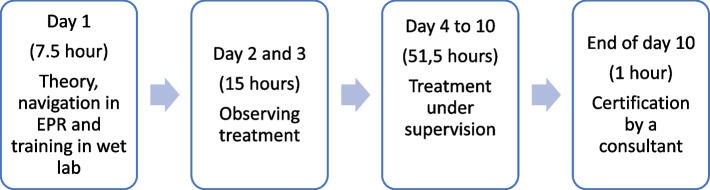
A 10-day training program to teach physicians and nurses how to administer injections. EPR = electronic patient record

Before the StO-study, all treatment were administered by physicians in training (residents) who spent six months of their residency administering injections. Before nurses were introduced to injection administration, a 10-day training program was designed (Fig. 1). Physicians followed the same training program as nurses except for the theoretical part and the navigation in the electronic patient record (EPR), meaning that their training program consisted of fewer hours spent by the teacher; six hours per physician versus 12 h per nurse. A re-certification for the nurses was arranged every other year with a refresher of theoretical and practical knowledge to maintain competence. The teacher’s wage cost during this re-certification was included in the training costs for nurses (4.5 h per nurse).

In addition to the teacher time cost, training costs also consisted of the time costs of physicians and nurses trained.

To estimate the training costs per injection, we divided the cost of training twenty physicians and six nurses by the expected number of injections administered in 10 years. This uneven number of physicians versus nurses occurs because physicians in training rotate every six months as part of their education to become a consultant (Table 3).

**Table 3:** Training cost (certification and re-certification) per injection

Group	Physician	Nurse
Certification costs per person ^a^	3964	3675
Re-certification costs per person ^b^	0	485
Certification and re-certification cost per person	3964	5616
Number of injections per person	2875	9583
**Certification and re-certification cost per injection**	**1,4**	**0,6**

^a^Theory, Wet lab, navigation in EPR, rehearsing injections and certification

^b^Do not apply to physicians because they rotate within the department more often than the two-year interval for re-certifications. EPR = electronic patient record. Costs are given in euros

#### Injection personnel costs

Wage cost per hour was multiplied by the number of hours per day (7.6 h for physicians and 7.5 for nurses). Wage cost per day was then divided by the average number of injections per day, giving the personnel costs per injection (Table 4).

**Table 4:** Hospital costs per injection for physicians and nurses

Cost categories	Physician		Nurse	
	€	%	€	%
Training costs	1.4	0.5	0.6	0.2
Injection personnel time costs	20.0	7.1	15.2	5.5
Clinical support costs	0.3	0.1	0.4	0.2
Support personnel time costs	36.3	12.9	36.3	13.1
Medicine	198.4	70.4	198.4	71.8
Equipment excluding medicine	19.1	6.8	19.1	6.9
Running expenses of operation premises	6.2	2.2	6.2	2.2
**Sum**	**281.6**	100.0	**276.1**	100.0

#### Clinical support costs

During the study period, certified physicians and nurses had the opportunity to seek advice from a consultant. The number of questions asked during the StO-study period was multiplied by the number of minutes used by the consultant and the consultant’s wage cost per minute, giving a total cost. This total cost was divided by the number of injections administered during the StO-study period, and this gave the clinical support costs per injection for physicians and nurses, respectively (Table 4).

#### Support personnel costs

At the injection clinic, a secretary worked 50%, and in addition, one nurse prepared patients and one nurse assisted injections. Wage costs for support personnel per year were divided by the number of injections administered per year, to find the support personnel costs per injection (Table 4).

#### Equipment

Information on which anti-VEGF medicine (ranibizumab, bevacizumab or aflibercept) was administered to each patient was obtained from the EPR, and the unit prices were obtained from the hospital pharmacy. The medicine costs for all patients were summed and divided by the number of injections to find the average medicine cost per injection. The equipment costs included the aggregated volume of disposable equipment at purchase price and the cost of cleaning reusable equipment, divided by the number of injections (Table 4).

#### Running expenses of the premises

The cost of the injection clinic premises was based on the area used and included expenses covering cleaning, electricity, and rental costs. Information about injection clinic area was obtained from the hospital's real estate department. (Table 2). The monthly cost was divided by the number of injections administered monthly to find the running expenses per injection (Table 4).

### Cost projections

To examine future cost consequences of task-shifting, we developed a simple model for projecting the number of injections per year for St. Olavs Hospital for the period 2022–27. For this purpose, the number of injections for 2014–21 was first obtained from the St. Olavs Hospital’s computer system (Nimes, LOGEX Healthcare Analytics, Oslo, Norway, 2022). For this period, we calculated the annual increase of injections per 1000 inhabitants in the St. Olavs population. We assumed this increase to represent an increase in the prevalence of retinal disease (patients per 1000 inhabitants) since injection per patient was presumed to be constant. The mean increase per year was 4.1%. Next, we used the number of injections administered in the StO-study to calculate age-group-specific injection prevalence for the year 2016, which is used as the reference year because this was the mid-year of the StO-study. We used five-year age groups from 30–89 years and 10-year from 90–99.

Population numbers and projections from Statistics Norway [6] were obtained to account for changes in the population composition over the next six years The age-group-specific injection prevalence was multiplied by forecasted population numbers in 2022–27 to project the number of future injections in the various age categories. To account for the increase in prevalence the estimated number of injections was multiplied by two alternative growth factors: four and two per cent.

To calculate potential hospital cost savings, the number of injections in 2014–27 was multiplied by the difference in the hospital cost per injection between physicians and nurses, respectively.

### Societal costs per patient

Societal costs per patient included hospital costs per injection (earlier described), other healthcare costs, transport costs and costs for caregiver’s time. Hospital costs per injection and transport costs per injection were multiplied by the number of injections for each patient to estimate the cost per patient.

Other healthcare costs were eye—and homecare costs. Eyecare costs were defined as costs for a consultation either at St. Olavs Hospital or at the patient’s local eye clinic (Table 2). The number of consultations was registered at each study visit. The standard cost for one consultation was multiplied by the number of consultations per patient to find the aggregated eyecare cost per patient.

Homecare costs was specified as costs for community-based homecare per year. The patient’s homecare use was registered at baseline and the end of the StO-study. The weekly costs were calculated by multiplying mean hours per week with the wage cost per hour of homecare personnel (Table 2). Aggregated homecare cost per patient was calculated, multiplying costs per week with the number of included weeks for each patient (Table 5).

Transport costs were calculated as the sum of travel costs (to eyecare and injections) for the patient and costs of the caregiver’s time of travelling plus waiting. The travel costs for the patient and the caregiver were calculated by measuring the distance and travel time from home to hospital and from home to eyecare with a web-based map [7]. Information on whether the patient travelled alone or with a caregiver, was registered at each visit in a questionnaire [8]. For patients who travelled alone, we calculated transport costs as the mean of the costs of taxi [9] and public transport (bus) [10], since information on the type of transport was not available for this patient category. For patients accompanied by a caregiver, we calculated the cost of using a private car (Table 2). Cost of caregiver consisted of costs for time spent on either injections or eyecare, and we estimated two hours for every visit. The total time was multiplied by the average wage for Norwegian employees in 2017 (Table 2).

### Statistical analyses

Descriptive statistics were used to present cost data. To compare the cost per patient between physicians and nurses, Mann–Whitney U tests were used. When calculating eyecare, homecare and transport costs, there were ≤ 0.01% missing values, and calculations were made for the complete cases. For other calculations, there were no missing values. P-values < 0.05 were considered statistically significant. Data preparation and statistical analyses were performed using IBM SPSS software 28.0 (IBM, New York, USA), and Microsoft Excel (Microsoft, Washington, USA 2013) was used for the cost projections.

### Ethics

Written informed consent was obtained from all patients. The study was approved by the Regional Committee of Ethics in Medical Research (2014/1719) and adhered to the Declaration of Helsinki. The study protocol was registered at ClinicalTrials.gov (NCT02359149).

## Results

### Hospital costs per injection

In Table 3 cost estimates for the training program for the certification and the re-certification are presented. On average, both personnel groups administered 23 injections per day during the StO-study. In total 57 500 injections over a 10-year period when injecting five days a week for 50 weeks per year. Training costs *per person* were lower for a physician compared to a nurse. However, over 10 years, a physician will be expected to administer 2875 injections, and a nurse 9583, resulting in higher physician training costs per injection.

The total hospital costs per injection were 5.5 € higher for physicians compared to nurses due to higher wage and training costs per injection (Table 4).

### Injection projections and possible future cost savings

**Fig. 2:**
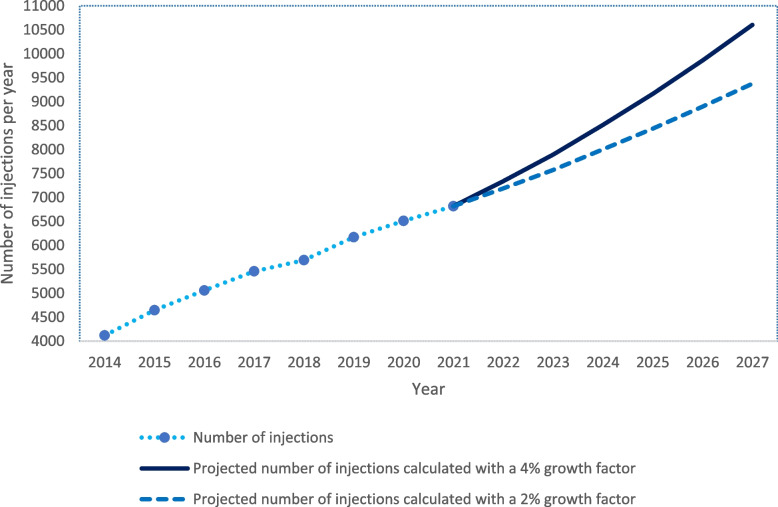
Number of administered and projected injections at St. Olavs Hospital

The observed number of injections for 2014–21 for St. Olavs Hospital and predicted annual injections for 2022–27 are shown in Fig. 2.

**Fig. 3:**
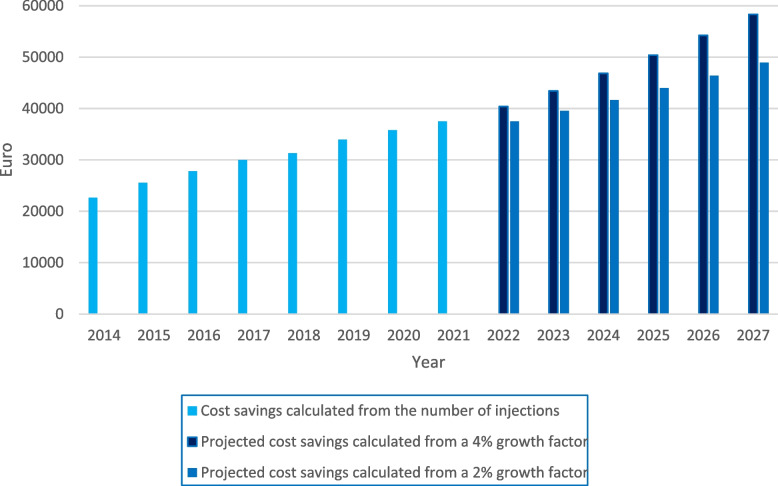
Estimated cost savings after task-shifting injections from physicians to nurses

An assumed annual increase in injection prevalence of four per cent implies an estimated 10 602 injections in 2027 compared to the observed 6817 in 2021. Applying a more modest growth factor of two per cent implies an estimated 9 374 injections in 2027. By combining annual injection numbers and the difference in hospital cost per injection in favour of nurses, we estimated annual costs saved for St. Olavs Hospital by task-shifting to nurses. The annual mean saving for the period 2017–21 was 33 705 € which in sum for the period was 168 527 €. The estimated total and potential cost savings for the period 2022–27 were 293 527 € (four per cent growth) and 258 017 € (two per cent growth), with an annual mean of 48 921 € and 43 003 €, respectively (Fig. 3).

### Societal costs per patient

Table 5 shows costs per patient for hospital costs, other healthcare cost, and transport costs for the one-year StO-study inclusion period. We found no statistically significant differences between the two groups, in any of the three societal cost categories.

**Table 5:** Societal costs per patient during one year of the St. Olavs study

							**Difference in mean**	
**Cost categories**	**Physician**			**Nurse**				***p*-value**
	Mean	Median	q25-q75	Mean	Median	q25-q75	Physician − Nurse	
*Hospital costs*	1946	1971	1408—2534	1895	1657	1381—2485	51	0.053
*Other healthcare*								
* costs*	*2057*	739	591—1182	2 275	887	591—1182	-218	0.942
* Transport costs*	960	766	253—1455	1 219	830	381—1478	-259	0.094
**Sum**	**4963**	**3695**	**2603—5525**	**5 389**	**3981**	**2710—5698**	**-426**	**0.403**

q25 and q75 are percentiles

*P*-values are calculated from Mann–Whitney U tests

Examining the *other healthcare cost* category, we observed that the use of homecare decreased by two minutes per week during the inclusion period in both the physician and the nurse group. Patients’ eyecare use increased by 29% and 32% during the StO-study for patients treated by physicians and nurses, respectively. In the transport costs category, we found that nurse patients travelled 3.3 km longer for each round-trip and travelled seven per cent points more often with a caregiver. In the physician group the use of a caregiver increased from 29% at baseline to 32% at the end of the study. For the nurse group, it decreased from 36 to 32%.

The mean hospital costs for all patients (physician and nurse group) were 1920 € (SD 748) per patient. Other healthcare costs per patient were 2169 € (SD 5531), and the transport costs were 1093 € (SD 1073). The societal costs per patient were 5182 € (SD 5777) for the StO-study year, which means that hospital costs per patient accounted for 37% of the societal costs.

## Discussion

In this study, the hospital cost per injection was estimated to be 5.5 € lower for nurses than physicians. Applying the estimated cost difference per injection and the projected number of injections, a potential hospital cost saving was found to be between 258 017 € and 293 527 € for the next six years if task-shifting the injections to nurses. We found no statistically significant difference in societal costs per patient between the two groups.

The hospital costs per injection were lower for nurses than physicians due to nurses’ lower wages. In St. Olavs Hospital, the wage of a certified nurse is 77% of the wage of a certified physician. The cost savings could be more substantial in countries where the wage gap is more prominent. For instance, in Singapore Teo et al. found a 61% reduction in cost per injection after task-shifting to nurses [11]. The cost categories that differed between physicians and nurses (training, injection- and support personnel costs) accounted for less than eight per cent of the total hospital costs per injection in the StO-study. This shows that the influence of time costs on the per injection cost was low, especially compared to the high medical costs, which account for over 70% of the total hospital costs. However, although anti-VEGF costs are high in Norway and other countries [12], it has been concluded that the improved vision associated with the treatment yields a substantial financial return on investment to patients and society [13].

The projected annual mean saving from task-shifting for St. Olavs Hospital from 2022–27 was between 43 003 and 48 921 € (two and four per cent growth). This amounts to half of the wage costs for a full-time nurse position per year. Hence, one can argue that the cost savings of task-shifting the injections to nurses are minor. However, more personnel trained equals more flexibility for the management and is also associated with higher treatment rates [14]. The physician resources can be reallocated to other urgent tasks, and a task shift like this is in line with WHOs recommendation to transfer tasks from a higher level of competence to a lower one [15]. It is also crucial for the visual outcome that patients receive treatment early after diagnosis and do not have to wait because of low capacity [16]. The Norwegian Ophthalmologic Society [17] has expressed concerns that the training and education of physicians do not meet the increasing demand. It is well known that in Norway, the rural areas have the most difficulties recruiting enough personnel. This highlights the need for a task shift that lowers costs and alleviates the injection burden. We have earlier reported that both experienced and less experienced nurses felt safe during training and treatment [18]. Several ophthalmology departments have task shifted injections from physicians to nurses with acceptable patient satisfaction [19–22] and results from an RCT indicate that such task-shifting is safe [4].

Several factors influence the future need for injections and thus the calculation of six-year cost projections. Increased awareness of retinal diseases in the population and increased use of advanced diagnostic imaging tools could increase the demand to some extent. Furthermore, novel drugs licensed for other retinal diseases [23, 24] like dry AMD [25] could increase the numbers substantially. On the other hand, development of longer-acting anti-VEGF injected as implants could lower the demand for injections [26]. We estimated that in 2027 the number of injections at St. Olavs Hospital will be between 9374 and 10 602. The upper estimate is calculated with a growth factor of four per cent, matching the mean of the last five years’ growth factor. We could have used a larger growth factor of seven per cent, matching the growth factor in 2021 at St. Olavs Hospital and used in an article from a UK tertiary hospital [27]. However, we chose to use the more modest estimate of two per cent because the UK hospital [27] reported that the growth factor appeared to be declining. They report a nearly 11-fold increase between 2009–19, which is more than double the increase at St. Olavs Hospital in the corresponding years. While the UK hospital used historical data to predict the number of future injections, we added population predictions to our model, making it more sensitive to age differences in the future.

The difference in societal costs per patient between the physician and nurse group was not statistically significant. Patients randomized to nurses travelled more often with a caregiver. However, the use of a caregiver decreased in the nurse group and increased in the physician group. There are more women in the nurse group, and there are reports that women more often utilize healthcare services and travel more with caregivers [28, 29]. This could be an explanation to the higher use of a caregiver in this group.

The mean travel cost per patient per year of all study patients was 1093 €, nurse and physician groups combined, a sum that is equivalent to 1/5 of the total societal cost per patient. The large number of patients receiving injection results in high travel costs for hospitals and society. For instance, in 2016 St. Olavs Hospital treated around 900 patients annually, with the estimated travel costs from the present study amounting to over 1 million euros. A study from Portugal found that the injection rates were negatively associated with travelling distances from home to hospital, meaning that patients that live farther off from the hospital are less likely to receive injections [14]. A Norwegian study observed the same geographical variation with higher injection rates in urban counties compared to rural ones [30] and suggested that travel distances to treatment may be one of the reasons why they observe geographical variations. The number of injections per 100 000 inhabitants in Portugal was 560 in 2018 [14]. In the population in Central Norway covered by St. Olavs Hospital, the corresponding number was 1818 (in the age range 0 – 105 + years), more than three times as many as in Portugal. However, even though St. Olavs Hospital is a university hospital in Norway’s third-largest city, the population distribution has a rural pattern, and many patients travel long distances for treatment (mean 57 km). This can explain the high travel costs that are reported in the present study.

The cost calculations had some limitations. We used a mean medical cost in the calculations since the actual medical costs of the three different medicines, are classified information. A further examination of medicine costs would have been of interest since the cost of various anti-VEGF-agents differ considerably and therefore choice of anti-VEGF-agent will likely affect hospital costs as such. However, when we compared injections by nurses versus physicians, we assumed equal distribution of anti-VEGF agents in both groups in accordance with randomization. It follows that a different choice will not affect the difference between nurses and physicians, only the level of hospital costs. We calculated time costs and clinical support costs for injections for the two groups, but available data did not enable the calculation of such costs for individual patients. Hence variation within groups remained unknown. A third limitation was the uncertainty when projecting the future number of injections. Pharmaceuticals are developing new long-acting anti-VEGF medicines [31], and alternative administration of anti-VEGF is also under construction [32] making the future projections less predictable. Since most eyecare consultations happened outside the hospital on different days than the injections, we might have overestimated the transport costs. In addition, we lacked detailed information on type of transport. Home-care costs will vary according to the patient population as a younger patient with unilateral diabetic macular edema or vein occlusion most likely need less help than an elderly patient with bilateral AMD. Our data mainly consisted of the latter patient category and hence the home care costs in this study might be high.

The strength of our study was the comprehensive calculation of hospital costs, covering most relevant cost categories. Moreover, the detailed calculation of injection costs like training, time costs and clinical support where data collected in the StO-study.

## Conclusion

To the best of our knowledge, the present study is the first to compare costs of physician- versus nurse-administered injections in a Nordic setting. Task-shifting the treatment to nurses provided a modest cost saving per injection for the hospital in addition to increased flexibility when reallocating physician resources from injections to other tasks. The societal cost per patient was not significantly different for physician- and nurse-administered injections. However, transport costs are high because of the long travel distances for patients in central Norway. Reducing transport costs by minimizing the number of clinical visits and organizing consultations and injections on the same day, may be a solution to reduce costs for society in the future.

## Data Availability

The dataset analysed during the current study is available from the corresponding author on reasonable request.

## Abbreviations

AMD: Age-related macular degeneration
anti-VEGF: Anti-vascular endothelial growth factor
DMO: Diabetic macular oedema
EPR: Electronic patient record
NTNU: Norwegian University of Science and Technology
NOK: Norwegian kroner
RVO: Retinal vein occlusion
StO study: St. Olavs study
